# Associations between *NBS1* Polymorphisms and Colorectal Cancer in Chinese Population

**DOI:** 10.1371/journal.pone.0132332

**Published:** 2015-07-17

**Authors:** Jing-Tao Li, Bao-Yuan Zhong, Hui-Hui Xu, Sheng-Yan Qiao, Gui Wang, Jing Huang, Hui-Zhen Fan, Hong-Chuan Zhao

**Affiliations:** 1 Department of Gastroenterology, China-Japan Friendship Hospital, Beijing, China; 2 Department of general surgery, First Affiliated Hospital of Gannan Medical College, Ganzhou, China; 3 Institute of Clinical Medicine, China-Japan Friendship Hospital, Beijing, China; 4 Department of Gastroenterology, The People’s Hospital of Yichun City, Yichun, China; Medical College of Soochow University, CHINA

## Abstract

As the central protein of the double strand breaks (DSB)-induced DNA repair pathway, *NBS1* participates in detecting the DSBs and plays an essential role in maintaining genomic stability. Single nucleotide polymorphisms (SNPs) in *NBS1* gene were commonly tested that associated with the susceptibility to multiple cancers, but the results remained controversial. Thus, we conducted two independent hospital-based case–control studies comprising 1,072 colorectal cancer patients and 1,263 controls to evaluate the association between four *NBS1* SNPs and colorectal cancer risk. The result showed that rs2735383C/G polymorphism in the 3’-untranslated region (UTR) of *NBS1* was significantly associated with risk of colorectal cancer using logistic regression (*P*<10^-4^). Furthermore, we observed that rs2735383CC genotype was associated with substantially increased risk of colorectal cancer (odds ratio=1.55, 95% confidence interval=1.27–1.94), compared with the rs2735383GC+GG genotypes. Further functional experiments demonstrated that the rs2735383C allele in the *NBS1* disrupted the binding affinity of has-miR-509-5p to the *NBS1* 3’-UTR in colorectal cancer cells, affecting the *NBS1* transcriptional activity and expression level. In conclusion, current evidence suggests that the rs2735383C/G polymorphism might contribute to the risk for colorectal cancer.

## Introduction

Colorectal cancer (CRC) is one of the most common malignancies leading cause of cancer-related deaths throughout the world[1]. In the last decade, great efforts have been focused on the development of new treatment modalities and diagnostic technologies, however, the incidence and mortality rate is significantly raising in an Asian population[2, 3]. In addition to environmental risk factors and lifestyle habits[4, 5], genetic alterations including single nucleotide polymorphisms (SNPs) in candidate genes which are involved in DNA repair pathway have been implicated in colorectal carcinogenesis[6–9].

DNA repair pathways play a key role in maintaining genomic integrity and preventing cells against cancer[10]. In contrast, it has become apparent that deficiencies of DNA double-strand breaks (DSBs) repair by carcinogens may lead to genomic instability and, consequently correlate with the development of cancer [11]. Two DNA repair pathways includes homologous recombination repair (HR) and non-homologous end-joining (NHEJ) pathways involved in human cells lead to damage repair by recruiting specific DNA repair molecules to the site of damage[12–14]. The recruitment of nuclear protein kinase ataxia telangiectasia mutated (ATM) to DNA DSBs is regarded as the critical step of both pathways in its activation and function. However, it has been recognized that ATM recruitment to sites of DSBs required and was facilitated by specific partner proteins meiotic recombination 11 homologue (MRE11), human RAD50 homologue (RAD50) and Nijmegen breakage syndrome 1 (NBS1)[15]. As a central player of the MRN complex, NBS1 directly interacts with H2AX to form nuclear foci at sites of DNA damage and triggers the subsequent steps in the DNA damage early response[15, 16]. Additionally, *NBS1* plays essential roles in the assembly of MRN complexes and directs MRN complexes coordinate DSBs to processing and repair [17, 18]. Therefore, genetic variants in *NBS1* gene may modulate DNA repair capacity, and affect the propensity to cause cancer. Accumulating studies have explored the associations between common polymorphisms in *NBS1* and human cancers risk [19–22]. For an example, one of the most commonly studied polymorphisms rs1805794G/C has been investigated associated with the susceptibility to multiple cancers [22–24]. Furthermore, the SNPs rs2735383C/G in the 3’-UTR of *NBS1* may influence the binding ability of microRNAs, thus may affect gene’s expression [22, 23]. However, limited information has been reported on the associations between these common *NBS1* polymorphisms and risk of CRC in Chinese population. Therefore, we hypothesized that common polymorphisms of the *NBS1* gene may associate with the risk of CRC.

Based on these hypotheses, we performed two hospital-based case-control studies to evaluate the possible influence of common polymorphisms (rs1805794G/C, 31129G/A, 924T/C and rs2735383C/G) on CRC risk in Chinese population.

## Materials and Methods

### Study subjects

All individuals were genetically unrelated ethnically homogenous Han Chinese, and eligible participants were 21–90 years of age at recruitment during the study period. For the discovery set, a total of 763 patients diagnosed CRC and 892 controls frequency-matched to the cases with regards to age (±5 years), sex and residential area were consecutively recruited from the People’s Hospital of Yichun City (Yichun, China) and China-Japan Friendship Hospital (Beijing, China). For the validation, 313 CRC patients and 371 controls frequency-matched to the cases with regards to age (±2 years), sex and residential area were consecutively enrolled from the China-Japan Friendship Hospital (Beijing, China). Among eligible participants, the recruitment rates were about 95% for patients and about 81% for controls. All patients hadn’t received any chemotherapy or radiotherapy and there were no age, stage, or histology restrictions. Tumor staging and pathological type were evaluated according to the 2002 American Joint Committee on Cancer staging system. The participation rate for controls was 89%. The clinical characteristics of all patients and controls of the discovery set and validation set are listed in Table 1. At recruitment, each participant was scheduled for an interview with an epidemiological questionnaire after giving a written informed consent and provided about 5ml blood sample for DNA analysis. The epidemiological questionnaire included demographic data, detailed information of smoking and alcohol consumption, family history of cancer and so on. This study were approved by the Medical Ethics Committee of The People’s Hospital of Yichun City and the Medical Ethics Committee of China-Japan Friendship Hospital of Beijing City.

**Table 1:** Distributions of characteristics among CRC patients and controls in Chinese populations used for pilot association study.

Characteristics	Discovery set				Validation set			
	Cases	^a^N(%)	Controls	^a^ N(%)	Cases	^a^ N(%)	Controls	^a^N(%)
**Mean age (range)**	56 (21–88)		56 (22–90)		56 (23–86)		57 (25–88)	
**Age (years)**								
≤60	425	(55.7)	498	(55.83)	183	(58.47)	209	(56.33)
>60	338	(44.3)	394	(44.17)	130	(41.53)	162	(43.67)
**Sex**								
Male	396	(51.9)	478	(53.59)	166	(53.04)	197	(53.1)
Female	367	(48.1)	414	(46.41	147	(46.96)	174	(46.9)
**Smoking Status**								
Positive	356	(46.66)	399	(44.73)	155	(49.52)	177	(47.71)
Negative	407	(53.34)	493	(55.27)	158	(50.48)	194	(52.29)
**Drinking Status**								
Positive	329	(43.12)	373	(41.82)	162	(51.76)	191	(51.48)
Negative	434	(56.88)	519	(58.18)	151	(48.24)	180	(48.52)
**Body mass index** (BMI)								
≤20	161	(21.1)	198	(22.2)	86	(27.48)	95	(25.61)
20<BMI<28	551	(72.22)	621	(69.62)	211	(67.41)	249	(67.12)
≥28	51	(6.68)	73	(8.18)	16	(5.11)	27	(7.28)
**Family history of cancer** ^**b**^								
Positive	63	(8.26)	80	(8.97)	27	(8.63)	33	(8.89)
Negative	700	(91.74)	812	(91.03)	286	(91.37)	338	(91.11)
**TNM stage**								
I	64	(8.39)			33	(10.54)		
II	330	(43.25)			124	(39.62)		
III	245	(32.11)			122	(38.98)		
IV	124	(16.25)			34	(10.86)		

^a^Number of cases/number of controls.

^b^Family history of cancer represents a history of all malignant tumors in first-degree relatives, which included fathers, mothers, brothers and sisters.

### Genotyping analysis

Based on the possibility of functional change of *NBS1* SNPs in the genome context, four *NBS1* SNPs (924T/C in 5’-UTR, rs1805794G/C in exon 5, 31129G/A in exon 10 and rs2735383C/G in 3’-UTR) previously reported in relation to disease outcomes including cancers were selected and analyzed [22, 25]. Genomic DNA was isolated from peripheral blood samples for each study subject using the Blood Genome DNA Extraction Kit (TaKaRa; Dalian, China). These four *NBS1* SNPs were genotyped by allele-specific MALDI-TOF mass spectrometry (Sequenom, San Diego, CA) as described before[26, 27] without the knowledge of the subjects status. Also, to confirm the genotyping results from the mass spectrometric analysis, we randomly selected 100 samples for direct sequencing, and the result was 100% concordant.

### Construction of reporter plasmids

**Fig 1:**
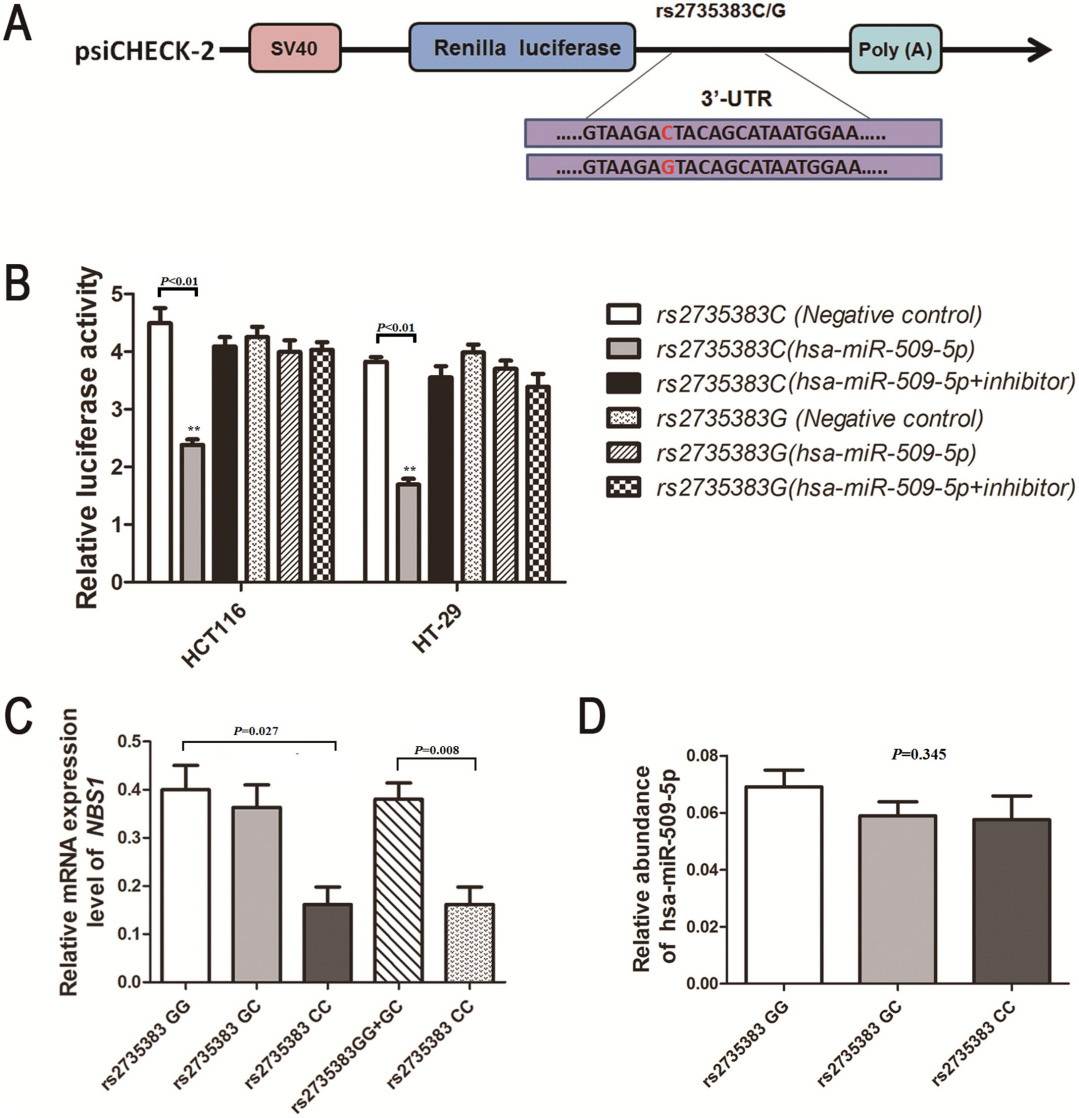
Schematic drawing of the luciferase reporter vectors (psi-CHECK2) contains *NBS1* 3’-UTR sequence with either the G or C at the rs2735383 locus. The luciferase activity of the rs2735383 variant allele on luciferase reporter bearing *NBS1* 3’-UTR co-transfected with or without hsa-miR-509-5p mimics or hsa-miR-509-5p inhibitors in HCT116 cells **(A)** and HT-29 cells **(B)**. Renilla luciferase activity was measured and normalized to firefly luciferase. Six replicates were carried out for each group, and the experiment was repeated at least three times. Data are mean±standard error of the mean; **P<0.01. **(C)** *NBS1* mRNA expression level in tumor tissue samples from colorectal cancer individuals with different rs2735383 variant alleles genotype (11 rs2735383GG, 13 rs2735383GC and 5 rs2735383CC); data are mean±standard error of the mean, normalized to *GAPDH*, *P* = 0.027. **(D)** hsa-miR-509-5p mRNA expression in 29 colorectal cancer tissues grouped by rs2735383 C/G genotypes. The hsa-miR-509-5p mRNA expression was calculated relative to expression of *U6* mRNA. Data are mean ± standard error of the mean, *P* = 0.345.

Since a significant association was observed for polymorphism rs2735383C/G and CRC risk, two reporter plasmids containing rs2735383G or rs2735383C allele were constructed to evaluate the effect of this polymorphism on its gene expression *in vitro*. The full-length of human *NBS1* 3’-UTR flanking SNP rs2735383 polymorphism was synthesized by the Genewiz Company and were then cloned into the psi-CHECK2 basic vector (Fig 1A). The two resulting constructs psi-CHECK2-*NBS1*-rs2735383C and psi-CHECK2-*NBS1*-rs2735383G were all re-sequenced to confirm the sequence and integrity of each construct’s inserts.

### Transient transfections and luciferase assays

Because the essential role of 3’-UTR in gene, we predicted the rs2735383C/G polymorphism in the 3’-UTR by our bioinformatics analysis (http://snpinfo.niehs.nih.gov/). The result revealed that the conversion from C to G of the 3’-UTR rs2735383 polymorphism cause new binding site of microRNA hsa-miR-629, has-miR-499-5p, and has-miR-509-5p. For the transient transfection assay, human colorectal cell lines, namely HCT116 and HT-29 were seeded at 1×10^5^ cells per well in 24-well plates (BD Biosciences, Bedford, MA). The luciferase assays were performed as described previously [28]. For luciferase activity analysis, 800ng psi-CHECK2-*NBS1*-3’-UTR vectors were cotransfected with 40pmol microRNA mimics (hsa-miR-629, hsa-miR-499-5p, and hsa-miR-509-5p) and with or without 40pmol inhibitors by Lipofectamine 2000 (Invitrogen, Carlsbad, CA) according to the manufacturer’s instructions. After transfection for 24h, cells were collected and renilla luciferase activity was measured with the Dual-Luciferase Reporter Assay System (Promega, Madison, WI) (Promega). Three independent experiments were done for each plasmid construct.

### Quantitative Real-Time PCR (qRT-PCR)

29 CRC tissues with different rs2735383 genotypes were subjected to investigate the correlation between the *NBS1* mRNA levels and rs2735383C/G polymorphism. Total RNA was isolated using the Trizol Reagent (Invitrogen Inc., USA) and reversed transcribed to cDNA using oligo-dT primer. We carried out ABI Prism 7500 sequence detection system to evaluate *NBS1* and *GAPDH* mRNA levels based on the SYBR-Green method. The latter was used as an internal quantitative control and *NBS1* expression level was normalized to *GAPDH*. The TaqMan MicroRNA Assays (Applied Biosystems) was used to detect hsa-miR-509-5p expression according to the manufacturers’ protocol and the universally expressed *U6* small nuclear RNA was used for an internal control.

### Statistical Analysis

The differences in the frequency distribution of selected demographic variables as well as rs2735383C/G allele and genotypes between cases and controls were assessed by the chi-square tests. The Hardy–Weinberg equilibrium (HWE) of the cancer-free controls genotype distributions was tested by a goodness-of-fit chi-square test. The strength of the association between the rs2735383C/G polymorphism and CRC cancer risk was estimated by odds ratios (ORs) with 95% confidence intervals (CIs) using unconditional logistic regression and adjusted for age and sex. Group’s comparison was analyzed by the Student’s t-test (two-sided). Stratified analysis was also performed by subgroups of age, sex or other characteristics (smoking status, alcohol drinking status, BMI, stage and family history) to evaluate the stratum variable-related ORs among the *NBS1* genotypes. One-way ANOVA were used to evaluate the differences in luciferase reporter activity and the effect of rs2735383C/G on the *NBS1* expression among the CRC patients. The statistical power was calculated by using the PS Software (http://biostat.mc.vanderbilt.edu/twiki/bin/view/Main/PowerSampleSize). All tests were two-sided by using the SAS software (version 9.1; SAS Institute, Cary, NC). Statistically significant level was defined as *P*<0.05.

## Results

### Genotypes and CRC risk

In the study, we genotyped four common polymorphisms (rs1805794G/C, 31129G/A, 924T/C and rs2735383C/G) of *NBS1* gene in 1076 CRC patients and 1263 healthy controls. The genotype frequencies of these polymorphisms in controls were consistent with the HWE (*P*>0.05 for all). The genotype distributions of *NBS1* polymorphisms of the cases and the controls in the discovery and validation set are presented in Table 2. A significant association with CRC risk was observed for rs2735383C/G but not for other polymorphisms in the two populations (*P*<10^−4^ for rs2735383C/G in the discovery set and *P* = 0.039 for rs2735383C/G in the validation set). As showed in Table 2, individuals with rs2735383CC genotypes were statistically significant higher risk than the carriers with rs2735383GG+GC genotypes (adjusted OR = 1.52; 95% CI = 1.20–1.99, *P*<10^−4^ for the discovery set and adjusted OR = 1.60; 95% CI = 1.11–2.37, *P* = 0.025 for the validation set). Furthermore, significant association was observed between rs2735383 polymorphisms and CRC risk when combined the two populations (*P*<10^−4^).

**Table 2:** Distribution of the two SNPs in *NBS1* in cases and controls associated with CRC.

Genotypes	Cases		Controls		Adjusted OR (95% CI)^a^	*P*_value_
	N	(%)	N	(%)		
**Discovery set**	**N = 763**		**N = 892**			
**rs1805794G/C**						
GG	218	(28.57)	278	(31.17)	1.00 (Reference)	
GC	399	(52.29)	451	(50.56)	1.13 (0.96–1.39)	0.308
CC	146	(19.14)	163	(18.27)	1.15 (0.81–1.56)	
**31129G/A**						
AA	88	(11.53)	90	(10.09)	1.00 (Reference)	
AG	336	(44.04)	421	(47.20)	0.82 (0.57–1.33)	0.962
GG	339	(44.43)	381	(43.71)	0.91 (0.59–1.43)	
**924T/C**						
TT	537	(70.38)	638	(71.52)	1.00 (Reference)	
TC	198	(25.95)	227	(25.45)	1.05 (0.84–1.38)	0.497
CC	28	(3.67)	27	(3.03)	1.23 (0.79–1.76)	
**rs2735383C/G**						
GG	225	(29.49)	315	(35.31)	1.00 (Reference)	
GC	364	(47.71)	433	(48.54)	1.08 (0.95–1.50)	**<10** ^**−4**^
CC	174	(22.80)	144	(16.15)	1.68 (1.28–2.25)	
GG+GC	589	(77.20)	748	(83.86)	1.00 (Reference)	
CC	174	(22.80)	144	(16.14)	1.52 (1.20–1.99)	<10^−4^
**Validation set**	**N = 313**		**N = 371**			
**rs2735383C/G**						
GG	102	(32.59)	138	(37.20)	1.00 (Reference)	
GC	146	(46.65)	180	(48.52)	1.07 (0.77–1.53)	0.039
CC	65	(20.77)	53	(14.29)	1.63 (1.07–2.61)	
GG+GC	248	(79.23)	318	(85.71)	1.00 (Reference)	
CC	65	(20.77)	53	(14.29)	1.60 (1.11–2.37)	0.025
**Pooled analysis**	**N = 1076**		**N = 1263**			
**rs2735383C/G**						
GG	327	(30.39)	453	(35.87)	1.00 (Reference)	
GC	510	(47.40)	613	(48.54)	1.13 (0.97–1.41)	<10^−4^
CC	239	(22.21)	197	(15.60)	1.68 (1.31–2.13)	
GG+GC	837	(77.79)	1066	(84.40)	1.00 (Reference)	
CC	239	(22.21)	197	(15.60)	1.55 (1.27–1.94)	<10^−4^

^a^Data was calculated by a logistic regression model with adjusted by age, sex, smoking status, alcohol use and family history of cancer. CI, confidence interval; OR, odds ratio.

We further performed the stratification analyses to evaluate the effects of rs2735383C/G genotypes on CRC risk according to age, sex, smoking status, alcohol consumption and family history of cancer in the discovery set. As showed in Table 3, a significantly increased risk of CRC associated with the polymorphism was more pronounced among subgroups of drinkers. Compared with the rs2735383GG and GC genotypes, CC genotype showed a 2.2-fold risk of CRC in drinkers (OR = 1.70, 95%CI = 1.51–2.88, *P* = 0.004). However, there was no statistically difference between the distributions of other selected variables and CRC risk.

**Table 3:** Stratification analyses of *NBS1* Polymorphism with CRC risk in the discovery set.

Variables	Cases (763)				Controls (892)				Adjusted OR (95% CI)^a^	*P*_value_ ^b^
	GG+GC(N)	(%)	CC(N)	(%)	GG+GC(N)	(%)	CC(N)	(%)	GG+GC *VS* CC	
**Age (years)**										
≤60	304	(39.84)	121	(15.86)	389	(43.61)	109	(12.22)	1.42 (1.07–1.93)	0.29
>60	285	(37.35)	53	(6.95)	359	(40.25)	35	(3.92)	1.92 (1.22–2.98)	
**Sex**										
Male	301	(39.45)	95	(12.45)	386	(43.27)	92	(10.31)	1.92 (1.30–2.80)	0.15
Female	288	(37.75)	79	(10.35)	362	(40.58)	52	(5.83)	1.33 (0.97–1.83)	
**Smoking Status**										
Positive	250	(32.77)	106	(13.89)	301	(33.74)	98	(10.99)	1.96 (1.31–2.91)	0.12
Negative	339	(44.43)	68	(8.91)	447	(50.11)	46	(5.16)	1.30 (0.95–1.81)	
**Drinking Status**										
Positive	199	(26.08)	130	(17.04)	284	(31.84)	89	(9.98)	2.09 (1.51–2.88)	**0.004**
Negative	390	(51.11)	44	(5.77)	464	(52.02)	55	(6.17)	0.95 (0.64–1.45)	
**Body Mass Index**										
≤20	129	(16.91)	42	(5.50)	157	(17.60)	41	(4.60)	1.24 (0.77–2.02)	0.43
20< BMI < 28	433	(56.75)	108	(14.15)	546	(61.21)	75	(8.41)	1.82 (1.32–1.50)	
≥28	27	(3.54)	24	(3.15)	45	(5.04)	28	(3.14)	1.43 (0.70–2.94)	
**Family history of cancer** ^**c**^										
Positive	31	(4.06)	32	(4.19)	42	(4.71)	38	(4.26)	1.14 (0.58–2.22)	0.28
Negative	558	(73.14)	142	(18.61)	706	(79.15)	106	(11.88)	1.69 (1.29–2.21)	
**TNM stage**										
I/II	318	(41.68)	76	(9.96)	748	(83.86)	144	(16.14)	1.62 (1.22–2.13)	0.59
III/IV	271	(35.52)	98	(12.84)	748	(83.86)	144	(16.14)	1.46 (1.03–1.96)	

^a^Adjusted for age, sex, BMI, smoking status, drinking status and family history of CRC as appropriate in a logistic regression model.

^b^ *P* value of the test for homogeneity between stratum-related ORs for *NBS1* (rs2735383GG+GC versus CC genotypes), the values of which were presented in bold, was defined as statistically significant.

^c^Family history of cancer represents a history of all malignant tumors in first-degree relatives, which included fathers, mothers, brothers and sisters.

### Effect of the *NBS1* rs2735383C/G polymorphism on transcriptional activity

We constructed luciferase reporter vectors by using the psi-CHECK2 vector with either the rs2735383C or rs2735383G allele and transfect transiently HCT116 and HT-29 cells with hsa-miR-629, hsa-miR-499-5p, and hsa-miR-509-5p, respectively. We examined whether the microRNA has an allele-specific effect on *NBS1* expression in CRC cells. The result showed that CRC cells transiently co-transfected hsa-miR-509 mimics and vectors containing the rs2735383C allele significantly decreased luciferase activity, compared with cells vectors containing the rs2735383G allele (*P*<0.05 in HCT116 cells and HT-29 cells; Fig 1B). However, no significant difference was found between this polymorphism and transcription activity with hsa-miR-629 and hsa-miR-499-5p in CRC cells. These data indicated that rs2735383C/G may affect the transcription of *NBS1* by influencing the binding site of microRNA to the *NBS1* 3’-UTR.

### Association of rs2735383C/G polymorphism with *NBS1* mRNA expression

We further examined the *NBS1* expression in 29 CRC tissues with different rs2735383C/G genotypes by qRT-PCR. As shown in Fig 1C, we found that patients with the rs2735383CC homozygote genotype had significantly lower *NBS1* mRNA levels (mean±standard error: 0.161±0.037) than patients with the GC (0.363±0.047) or GG (0.400±0.050; ANOVA test: *P* = 0.026) genotypes. Additionally, QRT-PCR was further used to examine whether hsa-miR-509-5p is constitutively expressed in CRC tissue samples. As shown in Fig 1D, hsa-miR-509-5p was constitutively expressed in CRC tissues; whereas there was no significant association between the background expression of hsa-miR-509-5p and the *NBS1* rs2735383C/G genotypes in tumor tissues collected from CRC patients (*P* = 0.345).

## Discussion

CRC carcinogenesis is multifactorial. Not only environmental risk factors but also genetic factors might play an essential role in the etiology of CRC. In this hospital-based case-control study, we investigated the role of four common *NBS1* SNPs in the susceptibility to CRC in Chinese populations. We found that hsa-miR-509-5p might bind tightly to 3’-UTR of *NBS1* containing the rs2735383C allele, negatively regulating the level of *NBS1*. Furthermore, in the following stratified analyses, it appeared that a high risk effect of this polymorphism was especially evident among subgroups of drinkers.

*NBS1* is a key regulator that participates in DSB repair by directing the MRN protein complex to the sites of DNA damage and stimulating its DNA binding and nuclease activity. The main functional domains of *NBS1* in DNA damage responses comprising the conserved forkhead-associated (FHA) domain and the breast cancer carboxy-terminal (BRCT) domain, which are important in the recognition and repair of aberrant DNA [29–31]. Numerous reports have highlighted the potentially crucial role of *NBS1*, an important part of MRN complex, in maintaining genomic stability and preventing the tumorigenic processes by recruiting repair and checkpoint proteins during the DNA breaks[32, 33]. Thus, the mutations of *NBS1* may affect the function of *NBS1* and protein-protein interaction, and further probably increase susceptibility of cancer. As one of the most commonly investigated polymorphisms, rs1805794G/C is located in the BRCT domain and influence the formation of a BRCA1-associated genome surveillance complex (BASC) though binding to BRCA1, which is responsible for detecting and repair of aberrant DNA structures[29]. Several lines of studies have investigated the association between the polymorphism and several cancers [22, 34–37]. However, the association between *NBS1* rs1805794G/C variant genotypes and cancer risk is not accordant in different cancers and ethnic group. For an example, P. Widlak found that rs1805794 variant genotypes were not associated with susceptibility of CRC in a Polish population [37]. Similarly, our present results are in accordance with the above study that the polymorphism rs1805794G/C is not associated with the risk of CRC in Chinese population.

To our knowledge, *NBS1* 31129G/A and 924T/C included in our study have rarely been investigated in human tumors [22, 25]. And we did not observe the relation between these two SNPs and CRC risk. As for rs2735383C/G polymorphism, it is located in 3’-UTR of *NBS1* gene. Several studies have investigated the rs2735383C/G polymorphism with cancer susceptibility. A meta-analysis based on thirteen eligible studies including 7561 cases and 8432 control subjects investigated that no significant association was found between the polymorphisms in *NBS1* gene and bladder cancer[38, 39], breast cancer[40], lymphoid malignancies [41]. Recently, it has been postulated that the above mentioned polymorphism rs2735383C/G could contribute to the development of lung cancer risk. More interestingly, another Chinese study among 575 lung cancer cases and 575 controls reported that the rs2735383C/G polymorphism was not associated with susceptibility of lung cancer [42]. In our study, rs2735383C/G polymorphism was significantly associated with CRC risk. Further stratification analyses showed that the increased risk associated with variant allele was more pronounced in drinking status. Further functional analysis showed that the nucleotide substitutions from G to C cause new binding site of microRNA hsa-miR-509-5p, thereby influencing the transcriptional activity of *NBS1* in *vitro*, which is consistent with the previous findings [24]. Taken together, these findings suggest that dynormal expression of *NBS1* resulting from the genetic variants in *NBS1* consequently affect the NBS1 protein and other repaired proteins interact in DNA repair processes, thus may modulate the CRC susceptibility.

This is the first study that we comprehensively investigated the associations between *NBS1* SNPs and the risk of CRC. We had a fairly large sample size for the case-control study. Moreover, the fact that the genotype frequencies among controls fit the Hardy–Weinberg disequilibrium law suggested the randomness of subject selection. And we have obtained a 91% study power (two-sided test, α = 0.05) to detect an OR of 1.52 for the rs2735383CC genotypes (which occurred at a frequency of 16.14% in the controls) compared with the rs2735383GC+GG genotypes in the discovery set, which may have enhanced the noteworthy of our finding.

In conclusion, our study suggests that polymorphism rs2735383C/G in the *NBS1* gene may be a genetic susceptibility factor for the risk of CRC in Chinese population. Moreover, our findings would require larger case–control studies and well-designed mechanistic studies to verify.
